# Phytochemical Characterization and Antioxidant/Antitumoral Potential of Coffee Silverskin: Comparative Insights with Green and Roasted Coffee

**DOI:** 10.3390/foods15142447

**Published:** 2026-07-09

**Authors:** Juliana A. Barreto-Peixoto, Cláudia Silva, Susana Machado, Maria Beatriz Prior Pinto Oliveira, Rita C. Alves, Fátima Martel, Nelson Andrade

**Affiliations:** 1 Associated Laboratory for Green Chemistry/Network of Chemistry and Technology (LAQV-REQUIMTE), Faculty of Pharmacy, University of Porto, R. J. Viterbo Ferreira, 228, 4050-313 Porto, Portugal; jpeixoto@ff.up.pt (J.A.B.-P.); claudiasilva@med.up.pt (C.S.); smachado@ff.up.pt (S.M.); beatoliv@ff.up.pt (M.B.P.P.O.); nandrade@med.up.pt (N.A.); 2 Unit of Biochemistry, Department of Biomedicine, Faculty of Medicine of Porto, University of Porto, Al. Prof. Hernâni Monteiro, 4200-319 Porto, Portugal; fmartel@med.up.pt; 3 Instituto de Investigação e Inovação em Saúde (I3S), University of Porto, R. Alfredo Allen, 208, 4200-135 Porto, Portugal

**Keywords:** coffee silverskin, green coffee, roasted coffee, by-product valorization, cancer cells, bioactive compounds, amino acids

## Abstract

Coffee is one of the most consumed beverages worldwide, and epidemiological studies suggest it may reduce the incidence of certain cancers. However, the anticancer potential of coffee silverskin (CS), the main by-product of coffee roasting, remains unexplored. This work aimed to valorize this bioresource by characterizing its bioactive composition and assessing its antioxidant and antitumoral potential. CS aqueous extracts were obtained through a fast and eco-friendly ultrasound-assisted extraction method and analyzed regarding caffeine, 5-caffeoylquinic acid, and amino acids (total and free). Their effects on cell growth, proliferation, viability, migration, lipid peroxidation, angiogenic factors, and cell cycle distribution were evaluated in pancreatic, breast, and colorectal cancer cell lines. Results were compared with those of green coffee (GC) and roasted coffee (RC) extracts. Despite presenting lower amounts of the bioactive compounds analyzed than GC and RC, CS extract exhibited a marked anticancer potential, surpassing the effects of GC and RC extracts in most assays. These findings position CS as a sustainable and high-value source of bioactive compounds with antitumoral potential, supporting biowaste valorization, circular economy, and sustainable production while contributing to health and well-being goals aligned with United Nations SDGs 12 and 3. Further studies are needed to clarify its mechanisms and in vivo efficacy.

## 1. Introduction

According to the World Health Organization (WHO), there are ~19 million new annual cases of cancer worldwide [1]. Chemotherapy is currently the most widely used anticancer treatment, but the occurrence of drug resistance, increased treatment costs, secondary toxicity, and adverse drug reactions [2] has prompted the search for alternatives. In this context, plant-derived bioactives hold considerable potential for exploration, as several epidemiological studies have found an inverse relationship between their consumption and the risk of developing certain cancers [3,4,5]. Additionally, several plant extracts have been shown to reduce proliferation rates, induce apoptosis, and alter cell cycle progression in various types of cancer cells in vitro. Importantly, plant-based extracts cause minimal side effects compared to chemotherapeutic drugs, and bioactives from plant sources can be combined with chemotherapeutic agents to minimize their side effects [6].

The increasing popularity and production of coffee in recent years (in 2023/2024, 10.7 million tonnes of coffee were produced worldwide) [7] has led to growing amounts of waste generated throughout the entire coffee chain, posing a serious environmental problem [8]. Coffee silverskin (CS), a thin tegument that surrounds the coffee beans, is the main by-product of coffee roasting. Its use as a potential bioactive ingredient for the development of novel functional products has emerged, contributing to environmental sustainability and economic recovery for coffee roasting companies [9]. CS contains a large number of bioactive compounds that are also present in green coffee (GC) and roasted coffee (RC), including caffeine and chlorogenic acids (CGAs), mainly 5-caffeoylquinic acid (5-CQA) [10]. These have been extensively studied and possess well-defined antioxidant and antitumoral potential [11,12,13]. In addition, CS is a good source of amino acids [14], which, besides playing pivotal roles in regulating key physiological processes, also exhibit antioxidant properties [15] and anticancer potential, although findings in this latter context remain conflicting [16].

Taking this into consideration, the present work aimed, for the first time, to analyze the effect of CS on different types of cancer cell lines (pancreatic, mammary, and intestinal), and to compare its effects with those of GC and RC. Furthermore, the extracts were chemically characterized regarding their caffeine and 5-CQA contents, as well as their total and free amino acids profiles. Our aim was to contribute to the valorization of this coffee by-product and its integration into circular economy scenarios and to highlight its potential in the complex context of cancer, in alignment with the United Nations Sustainable Development Goal (SDG) 12 (Responsible consumption and production) and SDG 3 (Good Health and well-being).

## 2. Materials and Methods

### 2.1. Standards, Reagents, and Materials

For chromatographic analyses, 5-caffeoylquinic acid (5-CQA), caffeine, individual amino acids, L-norvaline, the Amino Acid Mix Solution (certified reference material, Trace CERT^®^), and glacial acetic acid were all purchased from Sigma-Aldrich (St. Louis, MO, USA). The derivatization reagents (*o*-phthaldialdehyde/3-mercaptopropionic acid (OPA/3-MPA) and 9-fluorenylmethyl chloroformate (FMOC)) and borate buffer (0.4 N, pH 10.2) were obtained from Agilent Technologies (Santa Clara, CA, USA). HPLC-grade acetonitrile and methanol, and sodium azide (99%) were from Honeywell Riedel-de Haën^TM^ (Seelze, Germany), while di-sodium hydrogen phosphate anhydrous for analysis (≥99.0%) and di-sodium tetra-borate decahydrate 99–103% were from Merck (Darmstadt, Germany). All other reagents were of analytical-grade quality. Water was purified in a Milli-Q system (Millipore, Bedford, MA, USA).

For cell-based assays, ^3^H-thymidine ([methyl-^3^H]-thymidine; specific activity 79 Ci/mmol) was from GE Healthcare GmbH (Freiburg, Germany). Hydrochloric acid (HCl), triton X-100, and trichloroacetic acid (TCA) were purchased from Merck (Darmstadt, Germany). Acetic acid was from Scientific Laboratory Supplies (Nottingham, UK). Finally, antibiotic/antimycotic solution (100 U/mL penicillin, 100 mg/mL streptomycin, and 0.25 mg/mL amphotericin B), bovine serum albumin (BSA), fetal bovine serum (FBS), reduced nicotinamide adenine dinucleotide (NADH), sodium hydroxide (NaOH), sodium pyruvate, sulforhodamine B (SRB), tris-(tris(hydroxymethyl)-aminomethane hydrochloride), trypsin–ethylenediaminetetraacetic acid (EDTA) solution, doxorubicin hydrochloride; propidium iodide (PI), and hydroxyethylpiperazine-N0–2-ethanesulfonic acid (HEPES) were all obtained from Sigma-Aldrich (St. Louis, MO, USA).

### 2.2. Samples and Sample Preparation

Green robusta coffee beans from Cameroon (Africa) were kindly provided by BICAFÉ—Torrefação e Comércio de Café Lda (São Pedro da Cova, Portugal). The sample was divided into two equal parts. One of them was roasted (~210 °C, 10 min) and, afterwards, the RC beans and the detached CS were collected separately. All samples (CS, GC, and RC) were stored in a dry place and protected from light, being ground and homogenized before extraction.

The extracts were prepared by ultrasound-assisted extraction (UAE) as described by Peixoto et al. [10]. In brief, 500 mg of ground sample was extracted with 25 mL of distilled water for 10 min. For each matrix, eight technical extraction replicates (*n* = 8) were independently weighed from the homogenized ground sample and prepared, and, afterwards, extract replicates were combined, filtered, and freeze-dried (–80 °C, 0.015 mbar; Cryodos, Telstar, Barcelona, Spain). The extraction yields for CS, GC, and RC extracts were 15%, 29%, and 25%, respectively. These yields are comparable to those reported in previous studies for these matrices [17,18,19] and were calculated based on the mass of freeze-dried extracts relative to the initial samples mass (fresh weight), since it was not possible to determine their moisture content.

### 2.3. RP-HPLC-DAD Analysis of Caffeine and 5-CQA Contents

The freeze-dried extract (10 mg) was redissolved in deionized water (in triplicate) and centrifuged, and the supernatant was analyzed following the conditions described by Peixoto et al. [10]. The identification of compounds was performed by comparing retention times and UV absorption spectra with authentic standards.

### 2.4. RP-HPLC-FLD Analysis of Free and Total Amino Acids Contents

Free amino acid extraction was performed as described by Machado et al. [14]. Briefly, 10 mg of freeze-dried powder was extracted with 0.1 M HCl with magnetic stirring (20 min). Afterward, the mixture was centrifuged, and the supernatant was mixed with the internal standard (norvaline, 2 mg/mL).

Total amino acids were analyzed as previously described [14]. Briefly, alkaline hydrolysis was performed to quantify tryptophan, by mixing the freeze-dried powder (20 mg) with 4 M NaOH at 110 °C, for 6 h. Acid hydrolysis (for all the remaining amino acids) was performed by mixing the freeze-dried powder with 6 M HCl at 110 °C, for 24 h. The hydrolysates were centrifuged, and 50 µL of the supernatant was collected, neutralized, and mixed with the internal standard (norvaline, 2 mg/mL).

Extraction and hydrolysis were carried out in quadruplicate. Free and total amino acid profiles were then analyzed by RP-HPLC-FLD following the conditions previously described [14], after an automatic online derivatization with OPA/3-MPA and FMOC. Each amino acid was identified based on the retention time of the respective standards and quantified through the internal standard method.

### 2.5. In Vitro Cell Assays

#### 2.5.1. Cell Lines and Cell Culture

The following cell lines (ATCC, Manassas, VA, USA) were used: breast cell line MCF-7 (an estrogen receptor (ER)-positive human breast epithelial adenocarcinoma cell line; ATCC HTB-22; passage numbers 72–78); pancreatic cell line AsPC-1 (a human pancreatic cell line with high metastatic rate; ATCC CRL-1682; passage numbers 7–16); colorectal cell line HT-29 (a mucous-secreting human colorectal adenocarcinoma cell line; ATCC HTB-38; passage numbers 20–28); and colorectal cell line Caco-2 (a non-mucous-secreting human colorectal adenocarcinoma cell line; ATCC HTB-37; passage numbers 14–22).

Cells were maintained in a humidified atmosphere of 5% CO_2_–95% air and were grown in RPMI 1640 medium (catalogue #R6504, Sigma-Aldrich, St. Louis, MO, USA) supplemented with 2 mM L-glutamine, 1 mM sodium pyruvate, 10% heat-inactivated fetal bovine serum (FBS) and 1% antibiotic/antimycotic (AsPC-1 and MCF-7 cells), DMEM with 4.5 g/L glucose (catalogue #D5796, Sigma-Aldrich, St. Louis, MO, USA) supplemented with 2 mM L-glutamine, 10 mM sodium bicarbonate, 10% heat-inactivated FBS and 1% antibiotic/antimycotic (HT-29 cells) and MEM medium containing 5.55 mM glucose (catalogue #M-0643, Sigma-Aldrich, St. Louis, MO, USA) supplemented with 25 mM HEPES, 26.2 mM sodium bicarbonate, 15% heat-inactivated FBS and 1% antibiotic/antimycotic solution (Caco-2 cells). Culture medium was renewed every 2–3 days, and the cultures were split every 7 days. For the determination of cell viability, proliferation and culture growth, cells were seeded on 24-well culture dishes (2 cm^2^; Ø 16 mm; TPP^®^, Trasadingen, Switzerland) and used at 80–90% confluence. For cell cycle analysis, quantification of VEGF-A levels and determination of malondialdehyde (MDA) levels, cells were seeded on plates (21 cm^2^; Ø 60 mm; Corning Costar, Glendale, AZ, USA) and used at 100% confluence.

#### 2.5.2. Cellular Functional and Biochemical Assays

Cells were treated with CS, GC, or RC extracts for 24 h. The resuspension of freeze-dried extracts was performed as previously described [10] in culture medium without FBS (1:100 dilution). Controls were exposed to the same volume of water. In all assays, the extracts were tested at 1 mg/mL.

##### Determination of Cell Viability

Cell viability was evaluated by measuring the cellular leakage of lactate dehydrogenase (LDH) into the culture medium, as previously described [20]. LDH activity is expressed as the percentage of extracellular activity in relation to total cellular LDH activity.

##### Determination of Culture Growth

Culture growth was determined by the sulforhodamine B (SRB) assay, which reports on total protein content, as described by Andrade et al. [20].

##### Determination of Cell Proliferation

DNA synthesis rates were evaluated by quantification of incorporation of ^3^H-thymidine (µCi/mg total protein), as described by Silva et al. [21]. Results were normalized for total protein content.

##### Quantification of VEGF-A Levels

VEGF-A levels were quantified using a human VEGF-A ELISA kit (RAB0507; Sigma-Aldrich, St. Louis, MO, USA) according to the manufacturer’s instructions. The optical density at 450 nm was measured using a microplate reader (Thermo Fisher Scientific, Waltham, MA, USA).

##### Quantification of Malondialdehyde (MDA) Levels

MDA levels were quantified using a lipid peroxidation (MDA) assay kit (MAK085, Sigma-Aldrich, St. Louis, MO, USA) according to the manufacturer’s instructions. The optical density at 532 nm was measured using a microplate reader (Thermo Fisher Scientific, Waltham, MA, USA).

##### Cell Cycle Analysis

Changes in cell distribution across cell cycle stages were assessed by measurement of DNA content in the cells. After 24 h treatment, cells were centrifuged at 300× *g* for 5 min and fixed in 200 µL of 70% ethanol for 30 min at 4 °C. Next, cells were washed in PBS with 2% BSA and resuspended in 100 µL of propidium iodide (PI) solution (PI/Rnase, Immunostep, Salamanca, Spain) for 15 min at room temperature. Flow cytometry analysis was performed on a BD Accuri C6 flow cytometer (Becton, Dickinson and Company, Franklin Lakes, NJ, USA). Results were analyzed using FlowJo version 10.7.1 software (Ashland, OR, USA) and are expressed as the percentage of total cells arrested in each phase in the cell cycle.

##### Determination of Cell Migration

Cell migration rates were determined by a scratch injury assay, as previously described by Silva et al. [21]. Images were obtained at 0 and 24 h after the scratch, and quantification was performed using the ImageJ software version 2.14.0 (NIH, Bethesda, MD, USA).

##### Total Protein Determination

The protein content of cell monolayers was determined as described by Bradford [22], using human serum albumin as the standard.

### 2.6. Statistics

For chromatographic analyses, results were expressed as the mean ± S.D. (*n* = 3–4). Statistical analyses were performed using IBM SPSS 29.0 for Windows (SPSS Inc., Chicago, IL, USA), with one-way ANOVA, followed by Tukey’s HSD post hoc test; *p* < 0.05 was considered significant.

For cellular assays, data were expressed as the mean ± S.E.M. *n* represents the number of biological replicates, obtained across at least two independent experimental days. Statistical differences were assessed using Student’s *t*-test in GraphPad Prism 8.0 (San Diego, CA, USA); *p* < 0.05 was considered significant.

## 3. Results and Discussion

### 3.1. Chemical Composition of Extracts in Bioactive Compounds and Amino Acids

Knowing the chemical composition of plants/extracts is crucial for understanding their potential health benefits. Coffee and its by-products, including CS, are rich in bioactive compounds, mainly CGAs and caffeine, and also contain amino acids that contribute to their nutritional and functional properties [10,14,23]. Considering the reported antitumoral potential of these compounds [12,13], we quantified them in CS, GC, and RC, to clarify their contribution to the observed biological effects.

In this study, the selected bioactive compounds were recovered by ultrasound-assisted extraction (UAE), a green, cost-effective, efficient, and fast technique [8,24]. Acoustic cavitation enhances compound release with minimal structural damage, short extraction times, and reduced energy and solvent use, allowing water to be used as a non-toxic solvent [8,24]. Based on previously optimized conditions [8], UAE was applied to prepare CS, GC, and RC extracts. Their CGA and caffeine contents, as well as total and free amino acid profiles, were analyzed by PP-HPLC-DAD and RP-HPLC-FLD, respectively.

**Figure 1:**
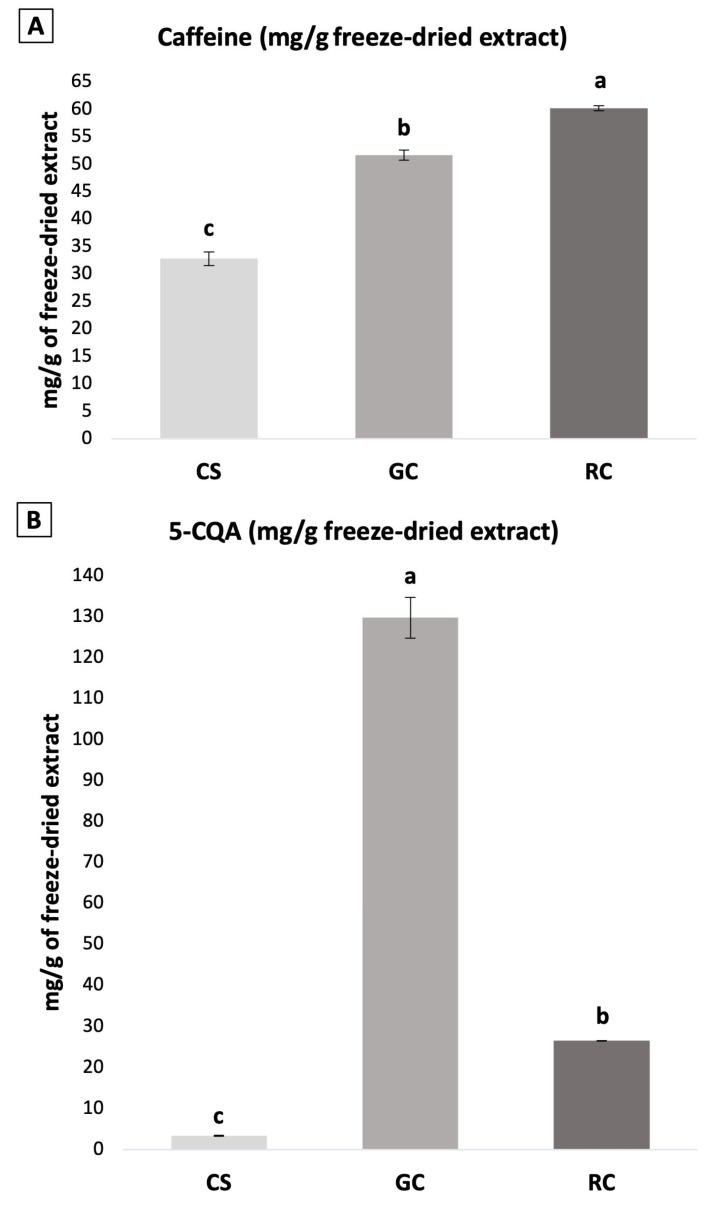
Phytochemical composition of coffee silverskin (CS), green coffee (GC), and roasted coffee (RC) extracts, expressed in mg/g of freeze-dried extract. (**A**) Caffeine contents of CS, GC, and RC extracts. (**B**) 5-CQA contents of CS, GC, and RC extracts. Results are expressed as mean ± S.D. Different letters within each graph represent significant differences between samples at *p* < 0.05, as assessed by one-way ANOVA, followed by Tukey’s HSD post hoc test.

Figure 1A,B depict the caffeine and 5-CQA contents, of the different extracts (in mg/g of freeze-dried extract). As expected, GC and RC showed significantly higher levels of both compounds compared with CS. Notably, the RC extract contained significantly more caffeine than the GC extract (Figure 1A). However, when the results were converted to mg/g of sample (Figure S1 of Supplementary Materials), no significant differences in caffeine contents were observed between GC and RC beans. This outcome was expected, as caffeine is a thermoresistant compound and its levels are not substantially altered by bean roasting. The lower amount of powder obtained after freeze-drying the RC extract compared with the GC extract (0.98 g vs. 1.14 g, respectively) indicates a higher concentration of caffeine per gram of freeze-dried material and further contributes to the similarity observed between the original samples after this conversion.

Regarding 5-CQA, significant differences were observed among samples: GC showed the highest content, followed by RC, with CS presenting the lowest values (Figure 1B). The lower 5-CQA content in RC compared with GC can be attributed to roasting, since CGAs are thermolabile and undergo degradation/transformation into lactones and melanoidins [25]. Similarly, CS, which was also exposed to roasting and detaches from the coffee during this process [8], showed significantly lower 5-CQA content than GC.

Overall, the caffeine and 5-CQA contents found in the present work are in accordance with previous studies. For example, Panusa et al. [26] also found significant differences between caffeine contents of GC and CS aqueous extracts (15.1 and 3.8 mg/g, respectively), which were slightly lower than those obtained in our study. In addition, a study corroborated the differences verified between GC and RC contents, with similar caffeine (GC: 12.3 to 19.9 mg/g; RC: 13.9 to 20.9 mg/g) and 5-CQA contents (GC: 27.2 to 39.2 mg/g; RC: 8.6 to 16.2 mg/g) [27] to those found in this study. The reported studies used water [26] and water:acetonitrile (95:5) [27] as extraction solvents and relied on conventional extraction methods that were more time- and solvent-consuming than the UAE approach herein applied. Therefore, this highlights the greater advantages of our extraction process (UAE), which proved to be faster, more efficient, and more environmentally friendly than conventional methods, using only a non-toxic solvent (water) in low amounts for the recovery of bioactive compounds from CS.

Besides the abovementioned phytochemicals, the amino acid composition (total and free amino acid profiles) of the extracts was also studied. Regarding total amino acids (Table 1), GC presented the richest amino acid profile, followed by RC and CS. Although the CS extract generally contained lower amounts of total amino acids than the RC extract, some amino acids (such as serine, arginine, and hydroxyproline) were present in higher levels than in the RC extract. Aspartic acid and glutamic acid were the main amino acids detected in all extracts, which is corroborated by the literature for the respective samples [14,25,28,29]. However, it is important to note that asparagine and glutamine were not detected in any extract, due to the acid hydrolysis employed, which deamidates these amino acids into aspartic and glutamic acids, respectively [14]. Therefore, the measured levels also take into account the conversion of asparagine and glutamine originally present in the extracts. All essential amino acids (histidine, threonine, valine, methionine, tryptophan, phenylalanine, isoleucine, leucine, and lysine) were detected in all samples (except lysine in RC), representing 36% of total amino acids present in the GC extract and 33% in both RC and CS extracts. Of these, branched-chain amino acids (valine, isoleucine, and leucine) were present in reasonable amounts, accounting for 15%, 17%, and 14% of total amino acids in the GC, RC, and CS extracts, respectively, as well as the non-essential amino acids serine (7%, 6%, and 8% in the GC, RC, and CS extracts, respectively) and arginine (7% in the GC and CS extracts, and 4% in the RC extract). These results show that, although present at lower amounts than in GC and RC, CS also provides all amino acids, including all essential ones, in proportions comparable to those of coffee.

Our CS extract presented significantly higher amounts of total amino acids than those found in a CS extract described by Iriondo-DeHond et al. [30], although the proportions were similar. There are other studies reporting total amino acid contents of CS, GC, and RC [14,28,29], but their results cannot be directly compared to ours as we examined the extract and not the sample. Even though the contents found in our extracts were lower than those reported in raw materials, the proportions of amino acids were similar.

Regarding the free amino acid profile (Table 2), the RC extract presented very significantly lower amounts of all amino acids compared to the CS and GC extracts. In turn, the GC extract showed the richest profile. The discrepancy between GC and RC samples, which are the same coffee beans before and after roasting, respectively, can be explained by the thermal processing to which the sample was subjected: it is well-described in the literature that roasting markedly destroys the free amino acids present in coffee beans, with an almost total loss [25,28,29], and that this degradation depends on the roasting conditions (temperature and time of roasting) [28,29]. Considering that our sample was subjected to a classic roasting procedure (210 °C, 10 min), these results for the RC extract were expected. It is of note that free amino acids, peptides, and proteins play a vital role in Maillard reactions that occur during the roasting process, which, as previously mentioned, are responsible for the formation of melanoidins (formed by reactions between their NH_2_ groups and the carbonyl group of reducing sugars; these reactions may also involve CGAs). Free amino acids are also involved in the formation of some hazard compounds, such as acrylamide, which results from the reaction between asparagine and reducing sugars [25,31], also explaining the significant lower amounts found in RC.

In sum, although less rich in free amino acids than the GC extract, the CS extract presented higher amounts than the RC extract and showed to be a source of free amino acids (2.5 mg of free amino acids/g of freeze-dried extract). Moreover, when comparing the free amino acid profiles of samples in more depth, some differences can be noticed. While for total amino acids the proportions of amino acids were similar between samples, in the case of free amino acids there were quite significant differences among the profiles of the different samples (Table 2).

### 3.2. Antitumoral Activity of Extracts

Several studies have reported an inverse association between coffee consumption and the risk of several types of cancer, including colorectal, hepatocellular, prostate, endometrial, brain, oral, and breast cancers [4]. CGAs and caffeine have shown anticarcinogenic properties, including the ability to inhibit cancer cell proliferation, tumor growth, angiogenesis, metastasis, and inflammation, as well as to induce apoptosis [12,13,32,33,34]. Most of these effects are linked to their antioxidant properties, but they further act on multiple molecular and cellular targets related to cancer [12,13,33]. Furthermore, the antioxidant and anticancer effects of amino acids have also been reported [15,16], although no consensus has been reached regarding their effect in the context of cancer. In fact, although some studies show that the restriction of amino acids might be an effective metabolic strategy for cancer therapy, recent studies concluded that amino acid supplementation can also be effective, particularly in cancer patients with cachexia. Therefore, amino acid-based interventions in cancer should be carefully individualized to suppress tumor growth while simultaneously supporting host metabolic needs [16].

So far, almost no studies have investigated CS in the context of cancer, although some potential anticancer effects (cytotoxic and antioxidant) have already been indirectly investigated [23,35]. The goal of this work was to investigate whether CS was able to affect neoplastic-related characteristics in different types of tumor cells in vitro, and to compare its effects with those of GC and RC extracts.

Based on the hypothesis that the CS, GC, and RC extracts would have antitumoral effects on pancreatic (AsPC-1), breast (MCF-7) and colorectal (HT-29 and Caco-2) cancer cell lines, we determined the consequences of a 24 h treatment with those extracts (1 mg/mL) on cell viability, culture growth, cell proliferation, cell cycle distribution, migration rates, angiogenic capacity, and oxidative stress levels.

#### 3.2.1. Effects on Cell Viability, Growth, and Proliferation

**Figure 2:**
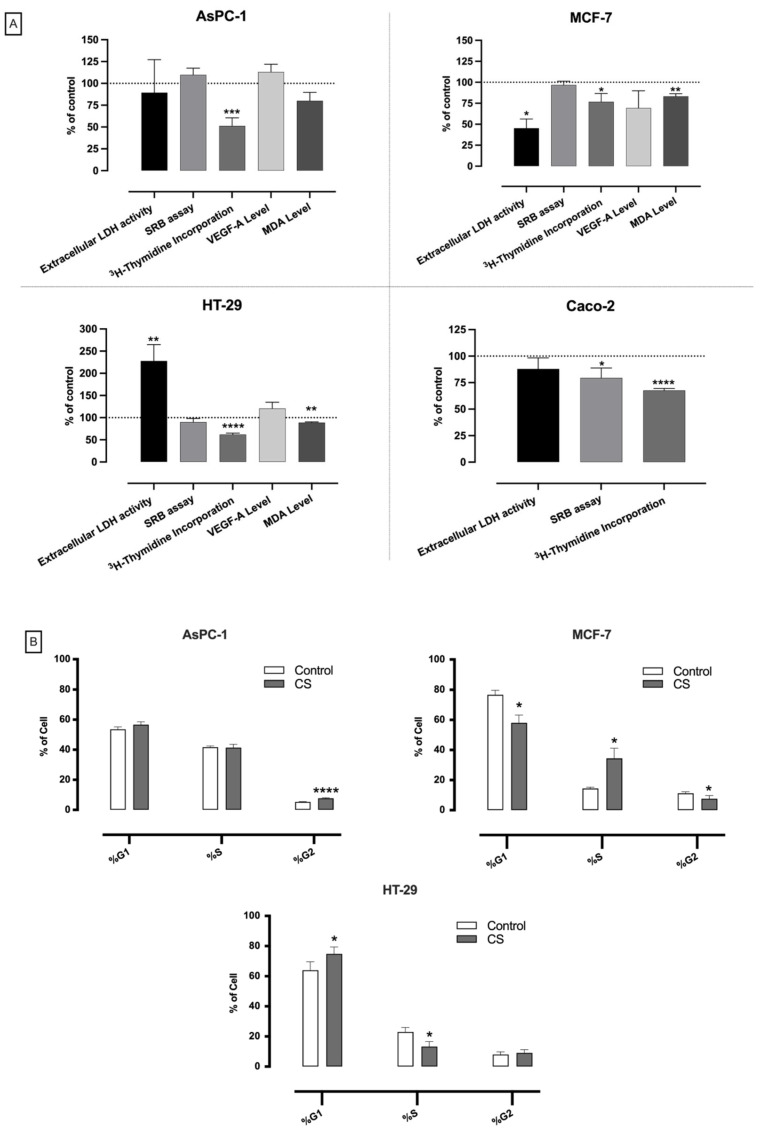
Effects of a 24 h treatment with coffee silverskin (CS) extract (1 mg/mL) on four different tumor cell lines (AsPC-1 (*n* = 6), MCF-7 (*n* = 6), HT-29 (*n* = 6), and Caco-2 (*n* = 7)). (**A**) Effect on cell viability (LDH assay), culture growth (SRB assay), cell proliferation (3H-thymidine incorporation), angiogenesis (VEGF-A assay), and oxidative stress levels (MDA assay). Results are presented in percentage of control (water). (**B**) Effect of the CS extract on the cell cycle (distribution of cells in phases G1, S and G2). Results are presented in percentage of cells (*n* = 6 per cell line). Means ± SEM are shown. * *p* < 0.05; ** *p* < 0.01; *** *p* < 0.001; **** *p* < 0.0001 significantly different from control (water) by Student’s *t*-test.

**Figure 3:**
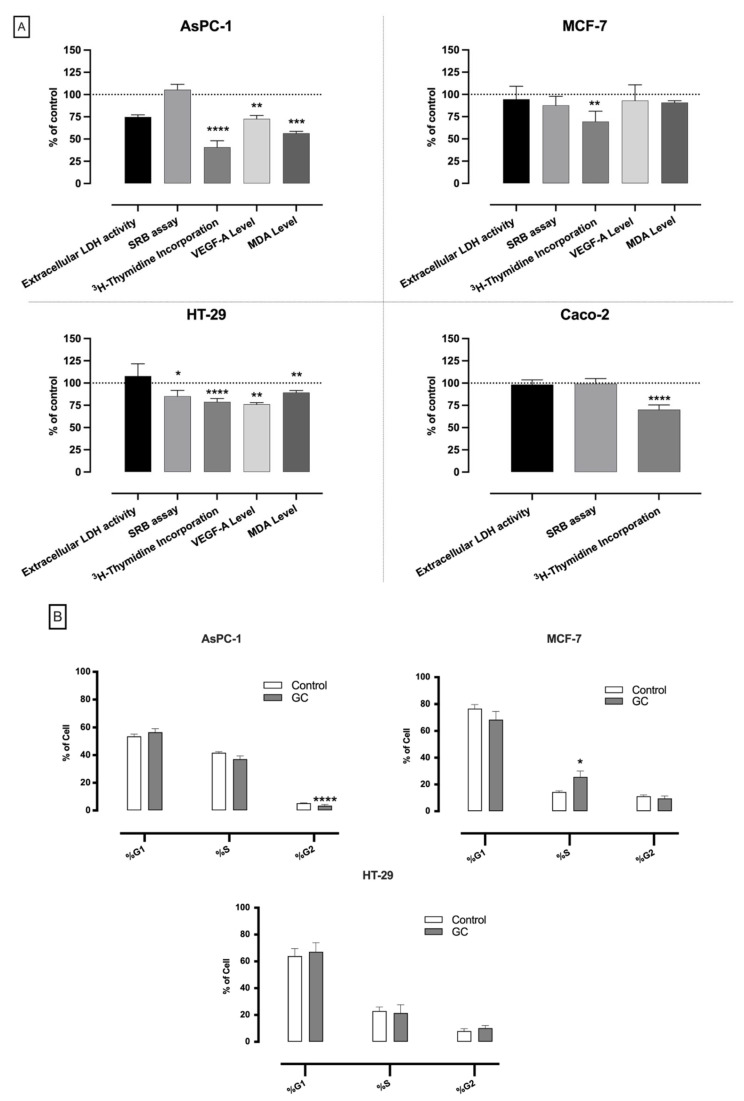
Effects of a 24 h treatment with green coffee (GC) extract (1 mg/mL) on four different tumor cell lines (AsPC-1 (*n* = 6), MCF-7 (*n* = 6), HT-29 (*n* = 6), and Caco-2 (*n* = 7)). (**A**) Effect on cell viability (LDH assay), culture growth (SRB assay), cell proliferation (3H-thymidine incorporation), angiogenesis (VEGF-A assay) and oxidative stress levels (MDA assay). Results are presented as percentage of control (water). (**B**) Effect of the GC extract on the cell cycle (distribution of cells in phases G1, S and G2). Results presented in % of cells (*n* = 6 per cell line). Means ± SEM are shown. * *p* < 0.05; ** *p* < 0.01; *** *p* < 0.001; **** *p* < 0.0001 significantly different from control (water) by Student’s *t*-test.

**Figure 4:**
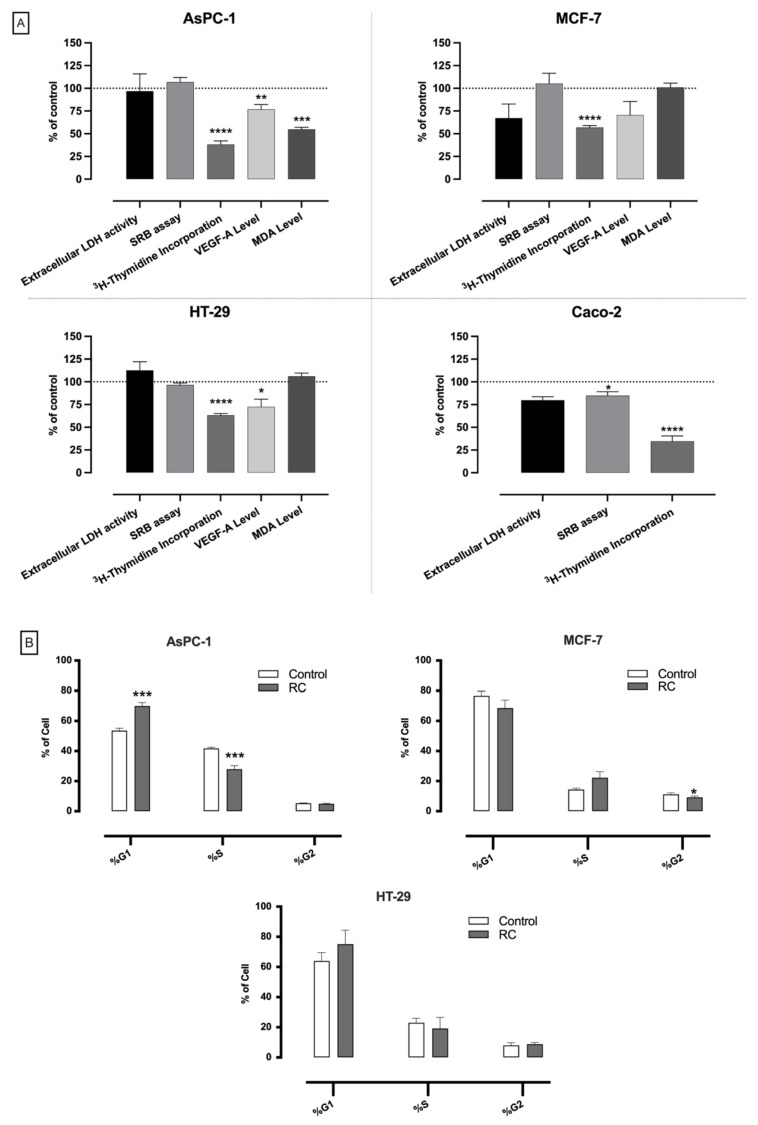
Effects of a 24 h treatment with roasted coffee (RC) extract (1 mg/mL) on four different tumor cell lines (AsPC-1 (*n* = 6), MCF-7 (*n* = 6), HT-29 (*n* = 6), and Caco-2 (*n* = 7)). (**A**) Effect on cell viability (LDH assay), culture growth (SRB assay), cell proliferation (3H-thymidine incorporation), angiogenesis (VEGF-A assay) and oxidative stress levels (MDA assay). Results are presented as percentage of control (water). (**B**) Effect of the RC extract on the cell cycle (distribution of cells in phases G1, S and G2). Results are presented in percentage of cells (*n* = 6 per cell line). Means ± SEM are shown. * *p* < 0.05; ** *p* < 0.01; *** *p* < 0.001; **** *p* < 0.0001 significantly different from control (water) by Student’s *t*-test.

Regarding viability results (extracellular LDH activity), CS had a cytotoxic effect on the HT-29 cell line only (Figure 2A). In contrast, neither GC nor RC were able to exert a cytotoxic effect on any of the three tumor lines studied (Figure 3A and Figure 4A). On the other hand, CS and RC reduced Caco-2 culture growth (SRB assay) (Figure 2A and Figure 4A), and GC reduced HT-29 culture growth (Figure 3A). These results agree with previous studies showing that CS, GC, and RC extracts (with caffeine and 5-CQA contents similar to those herein used) were generally not cytotoxic at 1 mg/mL in different cancer cell lines (PC-3 and DU-145 prostate cancer cells, HepG2 hepatoblastoma cells, and SW480 and SW620 colorectal cancer cells) [35,36,37], and that some reduction in culture growth was caused by RC and GC extracts in colorectal cancer cell lines [37]. Furthermore, several studies have evaluated the effects of 5-CQA on cell viability at concentrations comparable to those present in 1 mg/mL of our extracts (GC: 130 µg/mL, 367 µM; RC: 27 µg/mL, 75 µM; CS: 3 µg/mL, 10 µM). For example, Zeng et al. [34] found that a 24 h treatment with 5-CQA concentrations higher than 80 µM, 40 µM, and 10 µM significantly decreased the cell viability of MDA-MB-231, MDA-MB-453, and 4T1 breast cancer cells, respectively, while not affecting the non-cancer MCF-10 cell line. In another study, different concentrations of 5-CQA (1.25–80 µM) decreased HT-29 cell viability after a 96 h treatment period [38]. Moreover, Sadeghi Ekbatan et al. [39] showed that 5-CQA, caffeic acid, and a mix solution containing 5-CQA and its major microbial metabolites (caffeic acid, benzoic acid, and 3-phenylpropionic acid) reduced Caco-2 cell viability in a dose-dependent manner (50–1000 µM), with stronger effects in the mix solution, evidencing a synergistic activity between compounds. Interestingly, the results of the present study also suggest that the inhibitory effect of the different extracts on cell viability/culture growth cannot be solely attributed to 5-CQA, otherwise the GC extract, which presented the highest amounts of this compound, would have the most pronounced results in culture growth and cytotoxicity, which was not observed. Besides the metabolites studied by Sadeghi Ekbatan et al. [39], many other compounds in the extracts might present effects upon cell viability and culture growth, interacting with 5-CQA and resulting in different effects. In particular, caffeine, as a major bioactive compound found in coffee beans and silverskin, has also been widely studied regarding these effects. In general, other studies have shown that caffeine does not interfere with the viability of several cancer cell lines at concentrations of up to 500 µM [32,40], which are in the range of those detected in our extracts (GC: 52 µg/mL, 266 µM; RC: 60 µg/mL, 310 µM; CS: 33 µg/mL, 169 µM). In one of these studies, in addition to caffeine, other compounds commonly detected in coffee beans and silverskin (caffeic acid, 5-CQA, and kahweol) were tested and only kahweol was able to significantly reduce cell viability (≥50 µM) [40]. Furthermore, besides these compounds, we wondered about the potential role of amino acids upon the effects found. However, the existing literature is relatively scarce, and when available for some amino acids, they were tested at concentrations significantly higher than those herein detected and that were unlikely to be found in food. Just as an example, Corsetti et al. [41] found that a mixture of free EAAs possessed an antiproliferative effect and inhibited colon cancer growth by inducing apoptosis, both in vitro (CT26 cells) and in vivo (mice fed with diets enriched with EAAs), but the amino acids were tested in concentrations much higher (e.g., leucine: 676.5–1353 µg/mL in EAA mix solution) than those tested in the present study (e.g., leucine: 0.302 µg/mL in the GC extract). In general, considering our results and their comparison with other studies, it can be concluded that the cytotoxic and culture growth-inhibitory effects of the extracts depend on the concentration and the cell line used for the study, which presupposes that they act through different mechanisms of action in different cell lines. Moreover, our findings are corroborated by the cited literature, since cell viability of colorectal cancer cell lines seems to be the most susceptible parameter, both to extracts and to the compounds tested individually at the concentrations present in the extracts.

A ^3^H-thymidine incorporation assay was performed to ascertain cell proliferation. As shown in Figure 2A–Figure 4A, all the extracts demonstrated a significant antiproliferative effect in all the cell lines, being more pronounced in AsPC-1 cells. These results agree with a previous work where a concentration-dependent (0.3–1.2 mg of instant coffee powder/mL of culture medium) antiproliferative effect of an instant coffee powder was observed in the AH109A rat ascites hepatoma cell line. Interestingly, these authors also reported that the major compounds commonly found in coffee (caffeine, 5-CQA, caffeic acid, and quinic acid) had no inhibitory effects on AH109A proliferation, although no information on the concentrations tested was given [42].

Considering the results of LDH, SRB, and ^3^H-thymidine incorporation assays as a whole, it can be assumed that the GC, RC, and CS extracts exhibited a cytostatic activity without affecting cell viability and cell culture growth in AsPC-1 and MCF-7 cells. On the other hand, the GC and RC extracts presented significant antiproliferative effects by significantly reducing both DNA synthesis (^3^H-thymidine incorporation assay) and culture growth in HT-29 and Caco-2 cells, respectively, without exerting cytotoxic effects. Finally, the CS extract was the only one presenting significant effects on the three parameters evaluated in both intestinal cell lines (HT-29 and Caco-2). In Caco-2 cells, it reduced DNA synthesis and culture growth without cytotoxicity, indicating an antiproliferative effect through a cytostatic mechanism, while in HT-29 cells it reduced DNA synthesis and increased LDH levels, suggesting both cytostatic and cytotoxic effects.

#### 3.2.2. Effects on Cell Cycle Distribution

An antiproliferative effect is commonly associated with cell cycle arrest. For this reason, we decided to evaluate the effect of the extracts on cell cycle distribution (Figure 2B–Figure 4B). The results indicate that the three extracts, and, more markedly, CS, led to alterations in the cellular distribution in G1, S, and G2 phases. Indeed, the CS extract induced G2 (AsPC-1), S (MCF-7) or G1 (HT-29 cells) cell cycle arrest (Figure 2B), while the effects of GC and RC on cell cycle distribution were more discrete: GC caused only S-phase arrest of MCF-7 cells (Figure 3B) and RC caused only G1-phase arrest of AsPC-1 cells (Figure 4B).

These results perfectly agree with the results obtained with GC and RC extracts in breast [43], prostate [36], and colorectal [44] cancer cell lines, and further show that the CS extract maintains this ability. The effects are likely related to compounds such as 5-CQA, its derivatives (e.g., caffeic acid and 3-phenylpropionic acid), and caffeine, as these compounds, in the range of concentrations detected in our study, were previously found to induce G0/G1- and S-phase arrest in several cancer (colon (Caco-2 and HT-29) [38,39] and lung (NCI-H23) [32]) cell lines. The differences in the type of cell arrest induced by the extracts are most probably related to differences in the concentrations and proportions of these compounds. In addition, it is important to note that other bioactive compounds also present in coffee, such as cafestol [45] and trigonelline [46], have been reported to induce G1 cell arrest. Amino acids may also contribute to the effects found on the cell cycle. Nonetheless, as previously mentioned, the scarce studies assessing the effects amino acid on the cell cycle used concentrations much higher than those present in our extracts. Therefore, in further studies, it would be interesting to study the effects of amino acids at the concentrations present in the extracts in order to understand their potential contribution, in a more realistic scenario.

Considering the ^3^H-thymidine incorporation and cell cycle data, GC and RC generally presented greater DNA synthesis inhibition than CS, but milder effects on cell cycle, particularly in HT-29 cells, where cell cycle changes were not statistically significant. In turn, CS induced more pronounced alterations in cell cycle distribution, for all cell lines. These results suggest distinct molecular antiproliferative mechanisms. Namely, GC and RC mainly inhibited cell proliferation by inhibiting DNA synthesis during the S phase (altering cell cycle distribution to a lesser extent), while CS seemed to act on cell cycle checkpoints (G1/S and G2/M), inducing cell cycle arrest and indirectly reducing DNA synthesis.

The interference of coffee and CS extracts on cancer cell cycle was predictable, as Oleaga and colleagues reported that, of the 161 downregulated genes and 57 upregulated genes in HT-29 cells treated with instant (caffeinated) coffee, 25% of the overexpressed genes and 30% of the downregulated genes were related to the cell cycle [47]. Nevertheless, this is the first study reporting the effects of CS extract on the cell cycle of cancer cell lines (AsPC-1, MCF-7 and HT-29).

#### 3.2.3. Effects on Cell Migration

**Figure 5:**
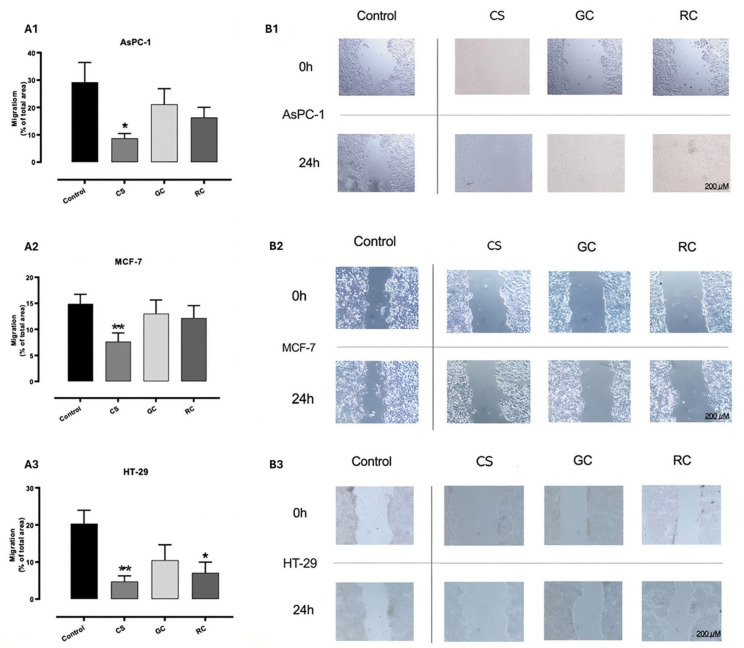
Effects of a 24 h treatment with coffee silverskin (CS), green coffee (GC), and roasted coffee (RC) extracts (1 mg/mL) on three different tumor cell lines (AsPC-1 (*n* = 8), MCF-7 (*n* = 12) and HT-29 (*n* = 8)) migration rates. (**A1**–**A3**) Effect on cell migration rates (scratch injury assay) on AsPC-1 (**A1**), MCF-7 (**A2**) and HT-29 (**A3**) cells. Results are expressed as Migration (% of total area). Means ± SEM are shown. * *p* < 0.05; ** *p* < 0.01 significantly different from control (water) by Student’s *t*-test. (**B1**–**B3**) Representative images of the pictures taken at 0 h and at 24 h after the scratch, obtained after exposure to each extract or the solvent (water), for AsPC-1 (**B1**), MCF-7 (**B2**) and HT-29 (**B3**) cells.

Regarding the effects on migration rates, the CS extract significantly reduced the migratory capacity of the three tumor cell lines, while the RC extract was able to decrease the migratory capacity of HT-29 cells only, and GC had no anti-migratory effect at all (Figure 5). Because our migration assay does not allow us to normalize for cell death and proliferation, the antiproliferative and/or cytotoxic effect of the extracts is expected to contribute to the decrease in migratory ability. Nevertheless, by comparing the anti-migratory and the antiproliferative/cytotoxic effects of the extracts (Figure 2A, Figure 3A, Figure 4A and Figure 5), it becomes evident that the CS extract possesses a clear anti-migratory effect in the three cell lines. To our knowledge, there are no previous studies reporting the anti-migratory potential of CS, and even studies on the anti-migratory effects of coffee (green and roasted) are relatively scarce. Namely, an anti-migratory effect of GC and RC extracts (presenting caffeine and 5-CQA contents similar to those found in our extracts and tested at similar concentrations in cells—0.5–1 mg/mL) on colorectal cancer cell lines (SW480, SW620, and HT-29) was found [37,48], which can be considered in line with our findings. In addition, several studies have already reported the anti-migratory capacity of compounds commonly found in coffee and CS, namely 5-CQA [34,37,49,50], caffeine [32,51], the diterpenes kahweol and cafestol [51,52], and even the amino acid arginine [53] in different cancer cell lines (breast, colorectal, lung, liver, prostate, and esophageal squamous cancer cell lines). Overall, considering that cell migration plays a crucial role in the initiation of cancer metastasis, a very complex event that often complicates the prognosis of several cancers [32], the significant anti-migratory effects of CS extract on the three cell lines show that this extract might possess anti-metastatic potential.

#### 3.2.4. Effects on Angiogenesis

Besides cell migration, angiogenesis plays a critical role in cancer growth and metastasis because solid tumors need a blood supply to be able to grow beyond a few millimeters in size. Tumors can actually induce blood supply by producing chemical signals that stimulate angiogenesis. VEGF-A is the key mediator of angiogenesis in cancer, and its increased production by the tumor greatly contributes to the ‘angiogenic switch’, where new vasculature is formed in and around the tumor, allowing it to grow exponentially [54]. Our results show that the CS extract did not have any effect on VEGF-A levels (Figure 2A). In contrast, both GC and RC caused a reduction in this parameter in AsPC-1 and HT-29 cell lines (Figure 3A and Figure 4A). These results suggest that components more abundant in the GC and RC extracts than in the CS extract are probably involved in this effect. The antiangiogenic properties of coffee components such as caffeine [55], 5-CQA [56,57], and other compounds [52] have been established in different cancer cell lines (e.g., breast, lung, and prostate cancer cell lines). However, to the best of our knowledge, the antiangiogenic potential of coffee extracts has previously been examined in only one study, and, in that work, a pro-angiogenic effect of an RC extract was described in the SH-SY5Y neuroblastoma cell line, through induction of VEGF expression levels [58]. Nonetheless, the authors did not associate this effect with any of the major compounds found in coffee extract (caffeine, 5-CQA, caffeic acid, and trigonelline), as they did not induce VEGF expression levels, but concluded that the coffee extract might present protective effects on neuronal disorders such as Alzheimer and Parkinson diseases [58]. Moreover, as far as we know, we assessed, for the first time, the antiangiogenic potential of CS.

#### 3.2.5. Effects on Oxidative Stress

Oxidative stress plays an important role in tumor initiation and progression and, in general, cancer cells have higher levels of reactive oxygen species (ROS) compared to healthy tissues, being under a basal state of oxidative stress [59]. Therefore, antioxidants might be able to reduce ROS and destabilize the oxidative stress status of cancer cells, leading to cell death. The effect of our extracts on oxidative stress levels was investigated by measuring lipid peroxidation (MDA) levels. The CS extract was able to significantly decrease MDA levels in MCF-7 and HT-29 cell lines (Figure 2A), whereas the GC extract reduced MDA levels in AsPC-1 and HT-29 cell lines (Figure 3A), and RC reduced MDA levels in AsPC-1 cells only (Figure 4A).

Many mechanisms are thought to underlie coffee’s antitumoral effects. Among these, the involvement of its antioxidant properties has been investigated. For instance, a GC extract (1–50 µg/mL) prevented cellular and macromolecular damage induced by *t*-BOOH in hepatocellular carcinoma cells (HepG2), reduced cytotoxicity and ROS levels, increased glutathione (GSH) levels, and reduced the levels of antioxidant enzymes (glutathione peroxidase (GPx) and glutathione reductase (GR)) [60]. Moreover, these effects were found to be associated with 5-CQA and 3,5-dicaffeoylquinic acid (3,5-DCQA), but not with caffeine [60]. In another study, both digested and non-digested coffee brew samples (0.25–0.50 mg/mL) were able to significantly decrease ROS intracellular levels in HT-29 cells, with a digested sample presenting higher antioxidant activity than non-digested, suggesting that digestion enhances the release of bioactive compounds with higher antioxidant activity [61]. In another study, GC and RC exhibited significant pro-oxidant effects when tested at high concentrations (2 and 4 mg/mL of RC extract and 4 mg/mL of GC extract) in HT-29 cells, which contributed to a concentration-dependent cytotoxic effect [44]. Overall, these results show that coffee samples may exert antioxidant or pro-oxidant effects depending on the concentrations tested, and in both cases, they may contribute to the anticancer effects.

Besides coffee samples, the antioxidant effects of CS extracts upon tumor cell lines have also been reported: Rebollo-Hernanz et al. [23] found that a CS extract (100 µg/mL) presenting similar amounts of 5-CQA and caffeine as those present in our CS extract was able to modulate palmitic acid-induced oxidative stress in HepG2 cells, by decreasing ROS and mitochondrial O_2_^•−^, preserving mitochondrial membrane potential, stimulating the nuclear factor (erythroid-derived 2)-like 2 (Nrf2) pathway, increasing the enzymatic activity of the antioxidant enzymes catalase and superoxide dismutase (SOD), and decreasing the enzymatic activity of NADPH oxidase. Moreover, the authors also found that the major bioactive compounds present in CS extract (caffeine, 5-CQA, and caffeic acid), tested at 50 µM, were also able to modulate oxidative stress. This highlights that the antioxidant ability of coffee and its by-products is related to their bioactives. Caffeine and CGAs are well-recognized antioxidants, capable of scavenging ROS and reactive nitrogen species (RNS), chelating pro-oxidant metals, and boosting antioxidant defenses [11]. Also, several amino acids can promote an antioxidant effect. In vivo studies and clinical trials have shown that amino acid supplementation may represent a co-therapeutic strategy in cancer by decreasing O_2_^•−^ levels, restoring GSH, and upregulating antioxidant defense genes (SOD1, SOD2, catalase, GPx1…) [16,62,63]. Although the free amino acids present in our extracts were lower than those described in the literature, limiting their contribution to the observed antioxidant effects, they may still partly account for the antioxidant activity observed in the different cell lines.

Overall, CS and coffee extracts exhibited antitumor effects in the cancer cell lines investigated. Of interest, the present report shows that, in vitro, the CS extract is as effective an antitumoral agent as its GC and RC counterparts, despite presenting significantly lower amounts of the studied bioactive compounds. In fact, these results were quite interesting and may have several interpretations. First, it is possible that other compounds, besides those herein quantified, might be present in higher amounts in CS extracts and contribute to its antitumoral effect. For example, CS is particularly rich in dietary fiber, including soluble dietary fiber, as well as in coffee melanoidins, which might even be conjugated to caffeine, CGA, and CGA derivatives [64,65]. Therefore, it is possible that these bioactive compounds may have also contributed to the antitumoral effects of CS extracts. Indeed, several studies have reported the potential antitumoral bioactivities of melanoidins and dietary fiber [65,66,67]. Although RC also contains melanoidins, their structure and composition may differ from those found in CS, potentially leading to them being less soluble and bioactive. Furthermore, the bioactive compounds present in the extracts might be present in different amounts and proportions, resulting in different interactions (e.g., synergistic, antagonistic, and additive) which may enhance or restrain the antitumoral effects found with the different extracts. These hypotheses should be investigated and validated in further studies.

Even though the present results demonstrate the antitumoral potential of CS extracts in comparison to GC and RC extracts, some aspects are worth noting. First, the concentration of the extracts used throughout the study (1 mg/mL) was selected as an initial screening concentration since no data are available regarding CS blood concentrations. This concentration falls within the range commonly employed in in vitro investigations of plant- and food-derived extracts, including those derived from CS, GC, and RC [17,35,37,42,44,48,65,68] and provided detectable biological activity under our experimental conditions. Therefore, it would be important in future to assess the bioavailability and blood levels of the main bioactive compounds of the extracts after their ingestion by humans, in order to test more physiologically relevant concentrations in in vivo studies and clinical trials. Second, it will be necessary to perform concentration–response experiments in order to determine IC50 values, thus allowing for a more robust pharmacological evaluation and comparison of the compounds. Finally, the effects of the extracts were evaluated exclusively in tumor cell lines, as the primary aim of this preliminary study was to evaluate and compare the effects of the three extracts across a panel of cancer cell lines representing distinct cancer types. Therefore, it will be important in future studies to determine the selectivity and therapeutic window (potential toxicity) of the extracts.

## 4. Conclusions

In this study, the chemical composition (caffeine, 5-CQA, total and free amino acids) and several antitumor-related effects of CS extracts were evaluated in pancreatic, mammary, and intestinal cancer cell lines and compared with GC and RC extracts. To our knowledge, this is the first report evaluating the effects of CS on cell proliferation, migration, angiogenic factors, and cell cycle. Interestingly, CS, similarly to GC and RC, possessed antitumoral properties against these distinct cancer cell lines. Mechanistically, although further investigation is needed, we were able to verify that the antitumoral effect of the three extracts is not directly related to their antioxidant effect and that CS appears to induce a more robust checkpoint arrest than GC and RC. CS, GC, and RC extracts obtained by ultrasound-assisted extraction were rich in 5-CQA, caffeine, and amino acids, and showed antitumoral activity in AsPC-1, MCF-7, Caco-2, and HT-29 cells. GC and RC contained higher levels of caffeine, 5-CQA, and total and free amino acids than CS (except for free amino acids, which were higher in CS than in RC). Notably, despite its lower levels of these compounds, CS exhibited marked antitumoral effects, in some cases even surpassing those of GC and RC, suggesting synergistic interactions among its bioactive compounds, as well as the presence and effects of yet unidentified compounds.

Overall, these findings indicate that, like GC and RC, CS is a valuable source of bioactive compounds with antitumoral activity, supporting the valorization of this coffee roasting by-product within circular economy strategies. Nevertheless, further research is needed to elucidate all bioactive components involved, clarify their mechanisms of action, assess bioaccessibility and bioavailability, investigate their effects upon non-tumor cell lines, and determine the real effects in an in vivo situation. This will guide a potential use and exploitation in nutraceutical and pharmaceutical formulations as chemopreventive and/or chemotherapeutic adjuncts.

## Figures and Tables

**Table 1:** Total amino acid contents of the different freeze-dried extracts (in mg/g of freeze-dried powder).

Amino Acid	CS	GC	RC
Aspartic acid	15.50 ± 0.45 ^b^	20.18 ± 0.95 ^a^	12.31 ± 0.54 ^c^
Glutamic acid	15.23 ± 0.75 ^c^	34.35 ± 3.04 ^a^	27.52 ± 0.94 ^b^
Asparagine	n.d.	n.d.	n.d.
Serine	7.34 ± 0.25 ^b^	11.63 ± 0.81 ^a^	5.99 ± 0.09 ^c^
Glutamine	n.d.	n.d.	n.d.
Histidine	1.25 ± 0.06 ^c^	4.84 ± 0.45 ^a^	2.43 ± 0.17 ^b^
Glycine	4.63 ± 0.27 ^c^	8.28 ± 0.79 ^a^	5.90 ± 0.24 ^b^
Threonine	6.32 ± 0.20 ^b^	9.72 ± 0.56 ^a^	5.87 ± 0.13 ^b^
Arginine	5.85 ± 0.21 ^b^	11.76 ± 1.06 ^a^	3.75 ± 0.10 ^c^
Alanine	5.95 ± 0.19 ^b^	9.89 ± 0.74 ^a^	6.43 ± 0.20 ^b^
Tyrosine	1.50 ± 0.15 ^c^	4.14 ± 0.47 ^a^	2.58 ± 0.11 ^b^
Valine	3.69 ± 0.21 ^c^	7.45 ± 0.69 ^a^	4.79 ± 0.18 ^b^
Methionine	2.73 ± 0.18 ^c^	3.61 ± 0.14 ^a^	3.12 ± 0.07 ^b^
Tryptophan	0.68 ± 0.02 ^c^	1.84 ± 0.08 ^a^	1.12 ± 0.04 ^b^
Phenylalanine	2.85 ± 0.20 ^c^	6.81 ± 0.63 ^a^	4.22 ± 0.15 ^b^
Isoleucine	4.23 ± 0.24 ^b^	6.84 ± 0.54 ^a^	4.80 ± 0.19 ^b^
Leucine	4.27 ± 0.25 ^c^	11.59 ± 1.10 ^a^	7.98 ± 0.16 ^b^
Lysine	3.13 ± 0.29 ^b^	8.97 ± 0.63 ^a^	n.d.
Hydroxyproline	0.97 ± 0.03 ^a^	0.58 ± 0.04 ^b^	0.52 ± 0.02 ^b^
Proline	2.51 ± 0.22 ^b^	5.98 ± 0.78 ^a^	3.21 ± 0.18 ^b^
Total amino acids	88.63	168.44	102.55
EAA	29.15	61.66	34.34
BCAA	12.19	25.88	17.58

Results are expressed as mean ± S.D. In each line, different letters denote significant differences between extracts (*p* < 0.05) assessed by one-way ANOVA, followed by Tukey’s HSD post hoc test. n.d., not detected. CS, coffee silverskin; GC, green coffee; RC, roasted coffee; EAA (essential amino acids): histidine, threonine, valine, methionine, tryptophan, phenylalanine, isoleucine, leucine, and lysine; BCAA (branched-chain amino acids): valine, isoleucine, and leucine.

**Table 2:** Free amino acid contents of the different freeze-dried extracts (in µg/g of freeze-dried powder).

Amino Acid	CS	GC	RC
Aspartic acid	585.44 ± 32.44 ^b^	1365.78 ± 72.30 ^a^	25.37 ± 0.79 ^c^
Glutamic acid	477.81 ± 8.72 ^b^	1530.28 ± 21.29 ^a^	4.98 ± 0.43 ^c^
Asparagine	157.36 ± 6.74 ^b^	2310.32 ± 55.55 ^a^	0.47 ± 0.04 ^c^
Serine	135.64 ± 1.32 ^b^	592.07 ± 30.19 ^a^	6.19 ± 0.51 ^c^
Glutamine	25.35 ± 0.52 ^b^	72.76 ± 3.47 ^a^	n.d.
Histidine	26.50 ± 1.90 ^b^	469.18 ± 18.66 ^a^	n.d.
Glycine	48.55 ± 2.14 ^b^	181.69 ± 6.04 ^a^	5.36 ± 0.28 ^c^
Threonine	62.24 ± 2.27 ^b^	161.98 ± 9.11 ^a^	6.28 ± 0.53 ^c^
Arginine	338.17 ± 15.64 ^b^	695.97 ± 37.17 ^a^	19.64 ± 0.68 ^c^
Alanine	234.80 ± 5.48 ^b^	1190.64 ± 28.45 ^a^	18.06 ± 0.71 ^c^
Tyrosine	24.39 ± 1.54 ^b^	412.30 ± 10.38 ^a^	1.54 ± 0.13 ^c^
Valine	91.38 ± 0.90 ^b^	364.07 ± 12.89 ^a^	1.22 ± 0.11 ^c^
Methionine	n.d.	61.74 ± 8.04	n.d.
Tryptophan	29.97 ± 3.37 ^b^	795.81 ± 34.22 ^a^	0.38 ± 0.16 ^c^
Phenylalanine	32.63 ± 0.93 ^b^	567.24 ± 10.83 ^a^	1.98 ± 0.21 ^c^
Isoleucine	79.85 ± 1.08 ^b^	289.70 ± 7.95 ^a^	4.01 ± 0.14 ^c^
Leucine	51.48 ± 1.64 ^b^	301.55 ± 9.19 ^a^	11.77 ± 0.35 ^c^
Lysine	30.67 ± 2.69 ^b^	315.77 ± 42.21 ^a^	n.d.
Hydroxyproline	8.39 ± 0.55 ^b^	37.75 ± 3.49 ^a^	n.d.
Proline	86.28 ± 3.48 ^b^	566.29 ± 60.05 ^a^	2.19 ± 0.04 ^c^
Total amino acids	2526.91	12,282.89	109.44
EAA	404.72	3327.04	25.64
BCAA	222.71	955.32	17.00

Results are expressed as mean ± S.D. In each line, different letters denote significant differences between extracts (*p* < 0.05) assessed by one-way ANOVA, followed by Tukey’s HSD post hoc test. n.d., not detected. CS, coffee silverskin; GC, green coffee; RC, roasted coffee; EAA (essential amino acids): histidine, threonine, valine, methionine, tryptophan, phenylalanine, isoleucine, leucine, and lysine; BCAA (branched-chain amino acids): valine, isoleucine, and leucine.

## Data Availability

The original contributions presented in this study are included in the article and the Supplementary Materials. Further inquiries can be directed to the corresponding author.

## Supplementary Materials

The following supporting information can be downloaded at https://www.mdpi.com/article/10.3390/foods15142447/s1, Figure S1: Phytochemical composition of coffee silverskin (CS), green coffee (GC), and roasted coffee (RC) extracts, expressed in mg/g of sample.

## Abbreviations

The following abbreviations are used in this manuscript:

5-CQA	5-caffeoylquinic acid
CGA	Chlorogenic acids
CS	Coffee silverskin
GC	Green coffee
GPx	Gluthathione peroxidase
GSH	Gluthathione
LDH	Lactate dehydrogenase
MDA	Malondialdehyde
RC	Roasted coffee
RNS	Reactive nitrogen species
ROS	Reactive oxygen species
RP-HPLC-DAD	Reverse-phase high-performance liquid chromatography coupled to diode-array detector
RP-HPLC-FLD	Reverse-phase high-performance liquid chromatography coupled to fluorescence detector
SRB	Sulforhodamine B
SOD	Superoxide dismutase
UAE	Ultrasound-assisted extraction
