# Supplementary material for: Phytochemical Characterization and Antioxidant/Antitumoral Potential of Coffee Silverskin: Comparative Insights with Green and Roasted Coffee
Source: Foods. 2026 Jul 9;15(14):2447. doi: 10.3390/foods15142447 (PMC13408514; doi:10.3390/foods15142447)
Supplement: Supplementary file 1 [file foods-15-02447-s001.zip › foods-4359621-supplementary.pdf]

Supplementary Materials

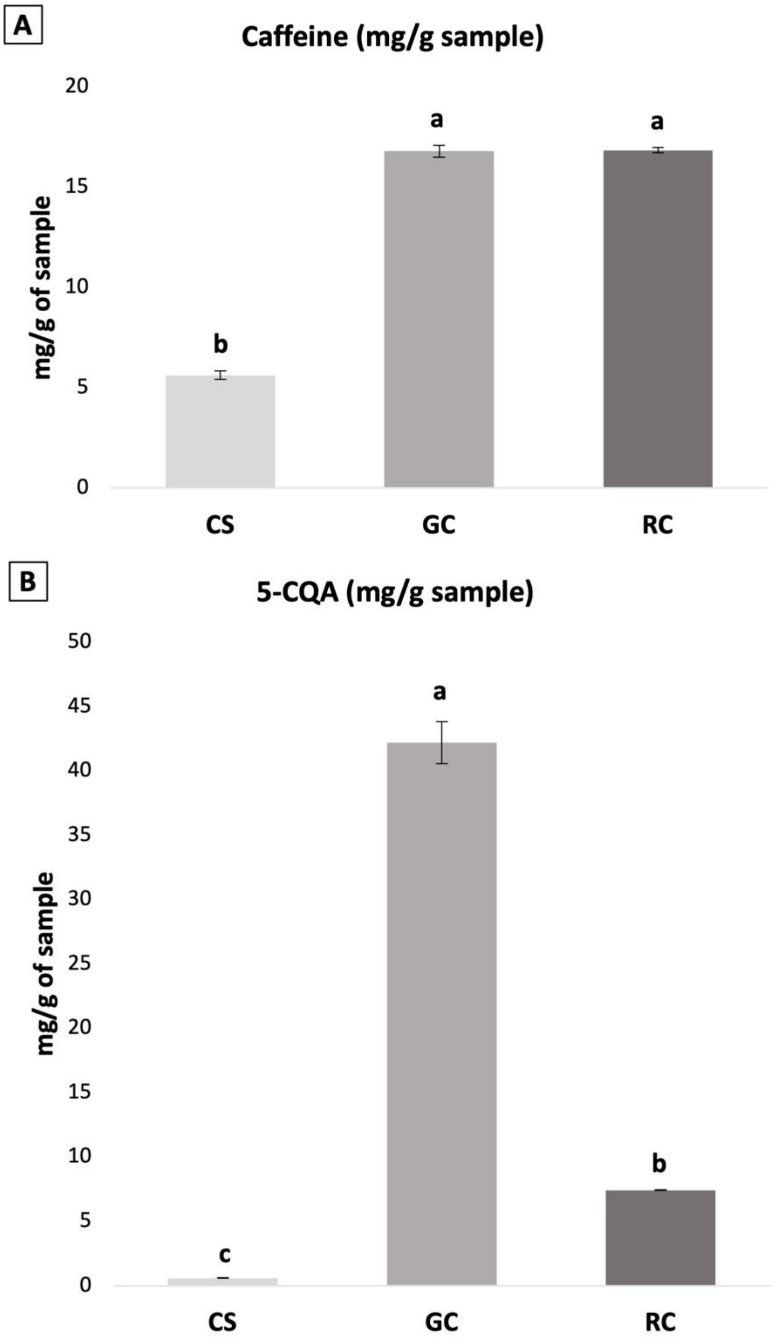

Figure S1. Phytochemical composition of coffee silverskin (CS), green coffee (GC), and roasted coffee (RC) extracts, expressed in mg/g of sample. (**a**) Caffeine contents of CS, GC, and RC extracts. (**b**) 5-CQA contents of CS, GC, and RC extracts. Results are expressed as mean ± S.D. Different letters within each graph represent significant differences between samples at $p < 0.05$.
